# Synchronous Multicentric Breast Cancer With Opposite Molecular Phenotypes: A Case Report and Therapeutic Considerations

**DOI:** 10.1155/crom/5068002

**Published:** 2026-08-24

**Authors:** Nelson Buelvas, Zully Acevedo

**Affiliations:** ^1^ Department of Surgery, Universidad del Sinú, Cartagena, Colombia, unisinu.edu.co; ^2^ Breast and Soft Tissue Surgery Department, IMAT Oncomédica-Auna, Montería, Colombia; ^3^ Pathology Department, IMAT Oncomédica-Auna, Montería, Colombia

**Keywords:** breast cancer, HER2-positive, immunohistochemistry, luminal breast cancer, multicentric carcinoma, tumor heterogeneity

## Abstract

**Background:**

Multifocal and multicentric breast cancers are increasingly diagnosed due to advances in imaging. Although some degree of molecular heterogeneity between tumor foci is recognized, most multicentric tumors share similar biological profiles. The presence of synchronous multicentric breast cancer with completely opposing molecular phenotypes remains exceptional.

**Case Presentation:**

We report the case of a 67‐year‐old woman diagnosed with synchronous multicentric breast cancer of the left breast. One tumor focus showed a HER2‐enriched phenotype (ER‐negative, PR‐negative, HER2‐amplified, and Ki67 60%), whereas a second spatially distinct focus (both confirmed on the surgical specimen) exhibited a luminal A phenotype (ER 100%, PR 1%, HER2‐negative, and Ki67 15%). Both lesions were confirmed by magnetic resonance imaging and independently biopsied. The patient underwent modified radical mastectomy and received adjuvant anti‐HER2 targeted therapy and endocrine therapy. At 22 months of follow‐up, she remains disease‐free.

**Conclusion:**

This case highlights the importance of comprehensive imaging assessment and independent molecular characterization of all suspicious tumor foci in multicentric breast cancer, as molecular discordance may have significant prognostic and therapeutic implications.

## 1. Introduction

Breast cancer is a biologically heterogeneous disease [1], with molecular classification playing a central role in prognostic stratification and therapeutic decision‐making [2]. Multifocal and multicentric breast cancers represent a subset of cases characterized by the presence of two or more synchronous invasive tumor foci within the same breast [3]. With the widespread use of advanced imaging modalities, particularly breast magnetic resonance imaging (MRI), the detection of these entities has increased [4].

Previous studies have shown that most multifocal and multicentric breast cancers share similar histological and molecular characteristics, supporting a common clonal origin with intramammary spread [5, 6]. Although intertumoral heterogeneity involving estrogen receptor (ER), progesterone receptor (PR), HER2 status, or Ki67 index has been described in a minority of cases [7–9], such differences are usually limited and rarely involve completely divergent molecular subtypes.

We present a case of synchronous multicentric breast carcinoma with discordant molecular subtypes between tumor foci. Rather than emphasizing the rarity of this biological phenomenon alone, this report highlights the clinical importance of comprehensive evaluation of every suspicious lesion in multicentric breast cancer. Independent biopsy and molecular characterization may directly influence systemic treatment decisions and should not be overlooked, even when one lesion appears to dominate the clinical presentation.

## 2. Case Presentation

### 2.1. Clinical Presentation

A 67‐year‐old woman was referred to our breast surgery service in April 2024 for oncologic evaluation of a previously diagnosed left breast carcinoma. The initial diagnosis had been established at an outside institution through her insurer′s early detection program, which had completed the diagnostic phase but did not provide definitive oncologic management.

At presentation, physical examination confirmed the presence of a palpable dominant lesion in the left breast at the 3 o′clock position. Notably, a second clinically suspicious lesion was also identified in the upper inner quadrant, around the 11 o′clock position, raising concern for multicentric disease.

**Figure 1:**
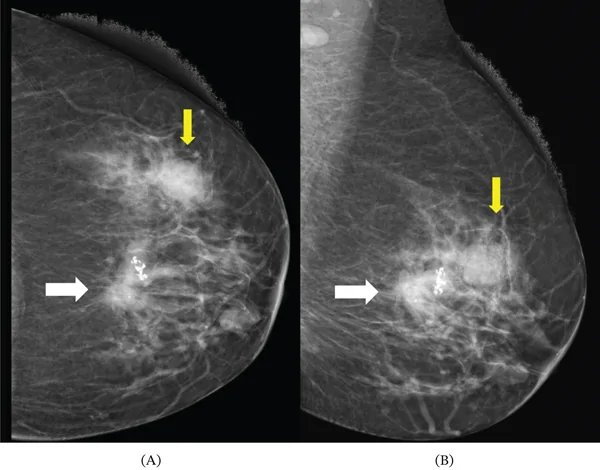
Mammographic evaluation demonstrating multicentric breast carcinoma with distinct imaging characteristics. (A) Craniocaudal (CC) view of the left breast showing two distinct lesions. The lesion indicated by the white arrow corresponds to an irregular, spiculated mass associated with calcifications, consistent with the luminal‐type tumor. The lesion indicated by the yellow arrow corresponds to a more circumscribed and less spiculated mass, corresponding to the HER2‐positive tumor. (B) Mediolateral oblique (MLO) view confirming the presence of both lesions with similar characteristics and spatial separation, supporting the diagnosis of multicentric disease.

Retrospective review of the outside mammogram supported this suspicion, revealing radiological heterogeneity between the two lesions (Figure 1). The dominant lesion at the 3 o′clock position (yellow arrow) appeared larger but less spiculated, whereas the lesion at the 11 o′clock position (white arrow) exhibited more pronounced spiculated margins and coarse calcifications despite its smaller size. This initial mammogram (April 2, 2024) described a dense irregular retroareolar‐deep mass with spiculated margins measuring 20 × 19 × 14 mm, along with two additional nodules in the left breast and ipsilateral axillary lymph nodes with cortical thickening (BI‐RADS 4C) (Figure 1). Subsequent breast ultrasound (April 12, 2024) confirmed the dominant lesion at the 3 o′clock position, corresponding to the HER2‐enriched tumor, with associated calcifications and parenchymal distortion, as well as a suspicious axillary lymph node. Core needle biopsy of this lesion demonstrated invasive ductal carcinoma, Grade 3.

Given the clinical and radiological suspicion of multicentric disease, breast MRI, additional biopsy of the 11 o′clock lesion, and staging studies were requested. MRI (May 6, 2024) confirmed the known HER2‐enriched lesion at the 3 o′clock position as an irregular enhancing mass with diffusion restriction (BI‐RADS VI). It also demonstrated the second lesion at the 11–12 o′clock position, corresponding to the luminal tumor, measuring approximately 15 × 12 mm, with irregular margins, internal calcifications, and contrast enhancement, considered suspicious for malignancy.

Ultrasound‐guided core needle biopsy of the 11 o′clock lesion (luminal tumor), performed at our institution on May 20, 2024, confirmed invasive ductal carcinoma, Grade 2. Immunohistochemical analysis of the biopsy demonstrated ER positivity in 70% of tumor cells, PR negativity, HER2 negativity, and a Ki67 proliferation index of 8%–14%, consistent with a luminal phenotype. Final immunohistochemical evaluation of the surgical specimen later demonstrated diffuse ER expression in 100% of tumor cells, PR positivity in 1%, HER2 negativity, and a Ki67 index of 15%, confirming the luminal molecular subtype.

In contrast, the 3 o′clock lesion (HER2‐enriched tumor) had initially been evaluated at an outside pathology laboratory, where the core biopsy demonstrated a poorly differentiated invasive carcinoma, Grade 3, negative for estrogen and PRs, HER2‐negative by immunohistochemistry, and a Ki67 index of 60%. Following surgery, repeat immunohistochemical evaluation of the surgical specimen demonstrated equivocal HER2 expression (2+). In accordance with current ASCO/CAP recommendations, fluorescence in situ hybridization (FISH) was subsequently performed and confirmed HER2 gene amplification, establishing the final diagnosis of a HER2‐enriched carcinoma.

These findings established the presence of two synchronous multicentric carcinomas with completely discordant molecular subtypes: a HER2‐enriched tumor at the 3 o′clock position and a luminal tumor at the 11 o′clock position.

Staging studies, including chest and abdominal computed tomography and bone scintigraphy, showed no evidence of distant metastasis. However, imaging findings suggested possible axillary nodal involvement.

### 2.2. Surgical Management and Pathological Findings

**Figure 2:**
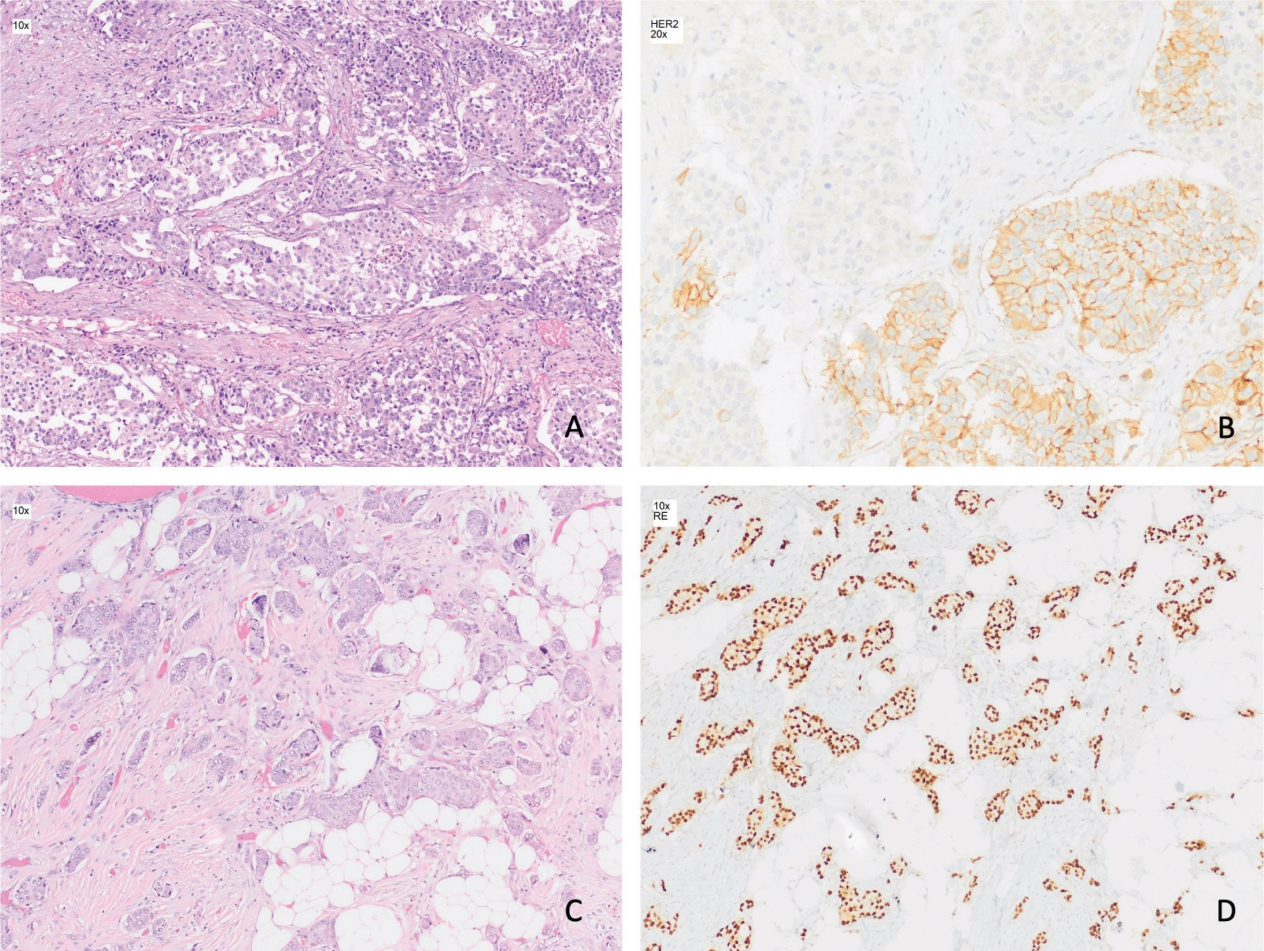
Histopathological and immunohistochemical characterization of multicentric breast carcinoma with discordant molecular profiles. (A) Hematoxylin and eosin (H&E) staining of the HER2‐enriched tumor (Block 5) demonstrating an invasive carcinoma of no special type, Grade 3, composed of solid nests of pleomorphic cells with increased mitotic activity and areas of central necrosis (10x magnification). (B) Immunohistochemistry for HER2 in the same lesion showing strong, complete membranous staining (3+), consistent with HER2 overexpression (20x magnification). (C) H&E staining of the second tumor focus (Block 6) demonstrating an invasive carcinoma of no special type, grade 2, composed of smaller, more uniform cells arranged in nests and single‐file patterns, with lower mitotic activity (10x magnification). (D) Immunohistochemistry for estrogen receptor (ER) in the second lesion showing strong and diffuse nuclear positivity, consistent with a luminal phenotype (10x magnification).

The patient underwent modified radical mastectomy with axillary lymph node dissection in June 2024. Final pathology confirmed two invasive carcinomas of no special type: a 4.7 cm Grade 3 tumor and a 2 cm Grade 2 tumor, both with negative surgical margins. None of the 36 dissected axillary lymph nodes showed metastatic involvement. Immunohistochemical analysis of the surgical specimen confirmed the molecular discordance between lesions, with HR negative and HER2 amplification in the dominant higher‐grade tumor and diffuse ER expression in 100% of tumor cells, PR positivity in 1%, HER2 negativity, and a Ki67 index of 15%, confirming the luminal molecular subtype in the second tumor (Figure 2).

### 2.3. Postoperative Course

Adjuvant systemic therapy was guided primarily by the biologically most aggressive component, and the patient received anti‐HER2 therapy with trastuzumab and pertuzumab. Given the presence of a hormone receptor‐positive tumor, endocrine therapy with tamoxifen was also initiated as part of multidisciplinary oncologic management.

At 22 months of follow‐up, the patient remains free of locoregional or distant recurrence, with no clinical or radiological evidence of disease.

## 3. Discussion

Multifocal and multicentric breast cancers represent a biologically heterogeneous subset of breast malignancies [3, 5, 6, 10]. Although traditionally considered the result of intramammary spread from a single primary tumor, increasing evidence suggests that molecular heterogeneity among tumor foci is not uncommon [11, 12]. Previous studies have reported discordance in hormone receptor status, HER2 expression, and Ki67 index in approximately 8%–24% of multifocal or multicentric breast cancers [7–9]. However, in most reported cases, such heterogeneity is limited to moderate variations within the same molecular spectrum, such as Luminal A versus Luminal B subtypes or differences in proliferative activity.

The present case illustrates an extreme and uncommon form of intertumoral heterogeneity, characterized by synchronous multicentric breast cancer with two spatially distinct invasive carcinomas exhibiting opposing molecular phenotypes: a HER2‐enriched tumor and a Luminal A tumor within the same breast [13]. This degree of molecular divergence—encompassing discordant hormone receptor expression, HER2 amplification status, and proliferative index—is rarely described in the literature and challenges the conventional assumption that multicentric tumors share a uniform biological identity.

From a biological perspective, two main hypotheses may explain this phenomenon [14]. One possibility is a common clonal origin followed by marked molecular divergence during tumor evolution. Alternatively, the coexistence of synchronous independent primary tumors arising within the same breast may account for the observed findings. In the present case, the striking differences in receptor status, HER2 amplification, Ki67 index, histological grade, and anatomical location make the latter hypothesis biologically plausible [15]. However, definitive discrimination between these mechanisms cannot be established based solely on clinicopathological and immunohistochemical findings. Molecular clonality analyses, such as next‐generation sequencing, comparative genomic hybridization, or copy number profiling, would be required to determine whether these tumors originated from a common ancestral clone or developed as independent primary neoplasms.

Importantly, regardless of the underlying biological mechanism, the clinical implications remain unchanged: each suspicious tumor focus should undergo independent pathological and molecular characterization whenever feasible, as biologically discordant lesions may directly influence systemic treatment decisions.

An additional and noteworthy aspect of this case is the radiological appearance of the two tumor foci. Despite being smaller in size, the Luminal A tumor demonstrated more pronounced spiculated margins on mammography, whereas the larger HER2‐enriched lesion appeared less spiculated, with more indistinct or obscured margins. This observation is particularly relevant, as spiculated morphology is traditionally associated with higher radiological suspicion and perceived aggressiveness [16]. However, imaging appearance does not always correlate with biological behavior [17].

This apparent discrepancy may be explained by differences in tumor growth patterns and stromal interactions among molecular subtypes. Luminal tumors, particularly Luminal A carcinomas, tend to grow more slowly and may induce a more pronounced desmoplastic stromal response, resulting in spiculated margins on mammography [18]. In contrast, HER2‐enriched tumors are characterized by high proliferative rates and more rapid, expansive growth, potentially allowing less time for organized fibrotic stromal reaction to develop and thus appearing less spiculated despite their aggressive biological behavior [19]. Although this pattern is not absolute, it underscores the limitations of relying solely on imaging features to infer tumor biology.

The clinical implications of molecular heterogeneity in multicentric breast cancer are significant. Current clinical practice often relies on the molecular characterization of the dominant or largest tumor focus to guide systemic therapy, an approach supported by generally high concordance rates in most series [10, 12]. However, in cases of marked molecular discordance, this strategy may lead to incomplete treatment. In the present case, independent biopsy and immunohistochemical assessment of each suspicious lesion revealed a Luminal A tumor that would have remained unrecognized had the additional lesion not been sampled. Failure to identify this component might have deprived the patient of the benefit of adjuvant endocrine therapy, highlighting that additional biopsies in multicentric disease may have direct therapeutic consequences beyond diagnostic confirmation.

Breast MRI complemented careful clinical examination by confirming the second lesion, which exhibited subtle but suspicious features that warranted further investigation. This emphasizes the importance of advanced imaging techniques combined with targeted biopsies to accurately define both disease extent and biological diversity [4, 20]. Comprehensive molecular characterization enabled a tailored treatment approach addressing both tumor components through anti‐HER2 targeted therapy and endocrine therapy.

This case also raises an important clinical dilemma: in multicentric breast cancer with discordant molecular subtypes, which tumor should define prognosis and guide systemic therapy? Although current staging systems assign tumor stage based on the largest invasive focus regardless of molecular subtype [21], emerging evidence suggests that biological aggressiveness may be more prognostically relevant than tumor size alone [22, 23]. In this patient, the HER2‐enriched tumor was both the largest lesion and the most biologically aggressive, simplifying therapeutic decision‐making. Nevertheless, a hypothetical inverse scenario would have posed a more complex challenge, further illustrating the limitations of anatomy‐based staging in biologically heterogeneous disease.

Surgical decision‐making in this case also warrants consideration. Current international guidelines generally recommend neoadjuvant chemotherapy combined with anti‐HER2 therapy for patients with clinically significant HER2‐positive breast cancer [24]. We acknowledge that this would have represented an appropriate therapeutic strategy had HER2 amplification been established preoperatively. However, management should ultimately be individualized. In the present case, biopsy‐confirmed multicentric disease had already established mastectomy as the definitive surgical procedure regardless of treatment response, eliminating the potential surgical benefit of tumor downstaging [25, 26]. Furthermore, the patient′s age, obesity, and comorbidities were also considered during multidisciplinary treatment planning [27]. Although delayed confirmation of HER2 amplification precluded consideration of neoadjuvant anti‐HER2 therapy, upfront surgery was regarded as a reasonable patient‐centered strategy in this specific clinical context. This individualized strategy reflects the importance of integrating tumor biology with patient‐specific factors in clinical decision‐making. This case also illustrates the practical consequences of delayed HER2 confirmation in real‐world multidisciplinary practice.

Axillary lymph node dissection was performed due to clinical and radiological suspicion of nodal involvement at diagnosis in the context of multicentric disease and an aggressive HER2‐enriched tumor. Although final pathology revealed no nodal metastases, the surgical approach reflected real‐world clinical judgment based on preoperative findings rather than retrospective outcome‐based reasoning.

In summary, this case highlights that multicentric breast cancer should not be assumed to represent a homogeneous disease entity. Differences in molecular subtype may not only influence prognosis and treatment but may also manifest as distinct imaging characteristics. Thorough imaging assessment, independent biopsy of suspicious lesions, and complete molecular characterization of all tumor foci are essential to avoid undertreatment and to optimize truly personalized therapeutic strategies. The rationale for the selection of tamoxifen rather than an aromatase inhibitor was not documented in the available medical records.

An additional limitation of this case relates to the delayed confirmation of HER2 amplification. The initial core biopsy, performed at an outside institution, classified the dominant lesion as hormone receptor‐negative and HER2‐negative. Only after surgical resection was HER2 immunohistochemistry re‐evaluated and interpreted as equivocal (2+), prompting FISH analysis that ultimately confirmed HER2 amplification. Consequently, neoadjuvant anti‐HER2 therapy was not considered during the initial treatment planning. Although this represents a limitation of the diagnostic pathway, upfront surgery remained an individualized and reasonable approach in this patient, given the presence of biopsy‐confirmed multicentric disease requiring mastectomy irrespective of treatment response, together with the patient′s age and comorbidities.

Although molecular discordance among multicentric breast carcinomas has been previously reported, the present case illustrates its practical implications in everyday clinical practice. Careful physical examination, critical reassessment of imaging findings, breast MRI, and independent biopsy of the second lesion resulted in identification of a biologically distinct luminal carcinoma that ultimately justified the addition of endocrine therapy. Had biological homogeneity been assumed based solely on the dominant lesion, this therapeutic opportunity might have been missed. Therefore, regardless of whether such discordance is uncommon or previously described, this case reinforces a practical diagnostic principle: each clinically or radiologically suspicious lesion should undergo independent pathological and molecular characterization whenever feasible, as the results may directly influence patient management.

## 4. Conclusion

Synchronous multicentric breast cancer may exhibit profound molecular heterogeneity, even with opposing biological phenotypes within the same breast. This case underscores the importance of comprehensive imaging assessment, independent biopsy of suspicious lesions, and complete molecular characterization of all tumor foci to avoid undertreatment and to optimize personalized systemic therapy. A biology‐driven approach is essential in the management of complex multicentric breast cancer presentations.

### 4.1. Learning Points

#### 4.1.1. Multicentric Breast Cancer Should Not Be Assumed to Be Biologically Homogeneous

Although most multicentric tumors share similar molecular profiles, marked intertumoral heterogeneity with opposing molecular subtypes can occur and may have relevant prognostic and therapeutic implications.

#### 4.1.2. Breast MRI Combined With Targeted Biopsies Plays a Critical Role in Accurately Characterizing Multicentric Disease

Independent sampling of each suspicious lesion is essential to define tumor biology and should be considered even when imaging features appear less aggressive.

#### 4.1.3. Molecular Characterization of All Invasive Tumor Foci May Directly Influence Systemic Treatment Decisions

In this case, identification of a Luminal A tumor allowed the appropriate use of adjuvant endocrine therapy, which would have been omitted had only the dominant HER2‐enriched lesion been evaluated.

#### 4.1.4. In Multicentric Breast Cancer With Molecular Discordance, Treatment Strategies Should Prioritize Biological Aggressiveness Over Tumor Size Alone

This case highlights the limitations of purely anatomy‐based staging systems and supports a biology‐driven, individualized approach to systemic therapy.

## Funding

No funding was received for this manuscript.

## Disclosure

The authors have read and approved the final version of the manuscript, had full access to all of the data in this study, and take complete responsibility for the integrity of the data and the accuracy of the data analysis. The authors affirm that this manuscript is an honest, accurate, and transparent account of the study being reported; that no important aspects of the study have been omitted; and that any discrepancies from the study as planned have been explained.

## Consent

Written informed consent for publication of clinical details and images was obtained from the patient.

## Conflicts of Interest

The authors declare no conflicts of interest.

## Data Availability

Data sharing is not applicable to this article as no datasets were generated or analyzed during the current study.
